# Analysis of facial ultrasonography images based on deep learning

**DOI:** 10.1038/s41598-022-20969-z

**Published:** 2022-10-01

**Authors:** Kang-Woo Lee, Hyung-Jin Lee, Hyewon Hu, Hee-Jin Kim

**Affiliations:** 1 Division in Anatomy and Developmental Biology, Department of Oral Biology, Human Identification Research Institute, BK21 FOUR Project, Yonsei University College of Dentistry, 50-1 Yonsei-Ro, Seodaemun-Gu, Seoul, 03722 South Korea; 2 Catholic Institute for Applied Anatomy, Department of Anatomy, College of Medicine, The Catholic University of Korea, Seoul, 06591 Republic of Korea; 3 Department of Materials Science & Engineering, College of Engineering, Yonsei University, Seoul, 03722 South Korea

**Keywords:** Musculoskeletal system, Bone, Cartilage, Muscle, Anatomy, Oral anatomy, Mandibular muscles, Medical imaging, Ultrasonography

## Abstract

Transfer learning using a pre-trained model with the ImageNet database is frequently used when obtaining large datasets in the medical imaging field is challenging. We tried to estimate the value of deep learning for facial US images by assessing the classification performance for facial US images through transfer learning using current representative deep learning models and analyzing the classification criteria. For this clinical study, we recruited 86 individuals from whom we acquired ultrasound images of nine facial regions. To classify these facial regions, 15 deep learning models were trained using augmented or non-augmented datasets and their performance was evaluated. The F-measure scores average of all models was about 93% regardless of augmentation in the dataset, and the best performing model was the classic model VGGs. The models regarded the contours of skin and bones, rather than muscles and blood vessels, as distinct features for distinguishing regions in the facial US images. The results of this study can be used as reference data for future deep learning research on facial US images and content development.

## Introduction

Facial anatomical structures are small and interconnected. Although these structures can be observed and distinguished well through dissection, the detection of the target muscle structure cannot be easily distinguished using imaging equipment such as magnetic resonance imaging (MRI) or computed tomography (CT). Distinguishing facial anatomical structures is important for detecting various diseases or performing cosmetic procedures such as botulinum neurotoxin^1–8^ and filler injections^9–11^.

While MRI and CT are considered standard medical imaging modalities that reveal high-resolution images of anatomical structures, potential disadvantages of these pieces of equipment include the need for radiation exposure for CT, elevated costs, and long analysis time^12,13^. As an alternative, ultrasonography (US), one of the most widely used imaging modalities, is considered to be a strong and omnipresent screening and diagnostic assessment tool for clinicians^1,4–6,8,14^. Over the decades, US has demonstrated several major advantages over other medical imaging modalities such as X-ray, MRI, and CT because of its convenience and cost-effectiveness^1,4–6,8,12,13^. However, US also has unique drawbacks, such as low image quality caused by artifacts, high dependence on practitioner experience, and differences in the manufacturers’ US system^12,13^.

To overcome these drawbacks, automated image analysis based on deep learning has recently been developed; however, there have been no attempts to apply this useful and smart method in the field of facial US anatomy^12,13^. The three major basic tasks of medical imaging, namely, classification, detection, and segmentation, are widely applied to different anatomical structures in medical US analysis, including the breast^15,16^, prostate^17,18^, liver^19^, heart/cardiac^20,21^, carotid^22^, thyroid^23^, intravascular^24,25^, lymph nodes^26^, kidney^27^, bone^28,29^, muscle^30^, nerve structure^31^. However, there have been no attempts to apply this useful and smart method in the field of facial US anatomy, which is the main cue of several non-invasive surgical procedures^32^.

Deep learning has rapidly developed in the automatic analysis of low- and high-quality medical imaging for diagnoses as well as image-based interventions^12,13^. Most of the classification models in the medical image field were created by using transfer learning from pre-trained models from ImageNet (Stanford Vision Lab, Stanford CA), which contains a wide variety of images ranging from faces to cats, cars, and mountains^33,34^. However, an intrinsic difference in image quality and complexity could affect deep learning performance and should be taken into special consideration in US applications^34^. The US images appear to have a significantly different image quality from that of ImageNet photos and other medical images^34^; therefore, it is crucial to evaluate several deep learning models before entering US images into deep learning algorithms and make US diagnoses and US-guided, non-invasive facial surgical procedures/therapies more objective, precise, and reliable.

Facial esthetic research has been conducted by using deep learning in facial aesthetic prediction^35–37^ and the facial rejuvenation recommendation system^38^. However, studies on the examination of the facial anatomical structures, which is helpful in diagnosing facial skin disease^39^, preventing iatrogenic side effects, and establishing the safest and most effective treatment plan, are few^1,4,6,8,9,32,40,41^. Moreover, several previous deep learning models have not yet established which model is acceptable to classify the facial US images and how many data sets are needed, even though the anatomical information is crucial for some clinical tasks such as deciphering facial structures of US images before a procedure. Therefore, we aimed to estimate the value of deep learning for facial US images by assessing the classification performance for facial US images through transfer learning using current representative deep learning models and analyzing the classification criteria.

## Materials and methods

All experimental procedures in this study were performed in accordance with the Declaration of Helsinki of the World Medical Association (version of October 2013). The study was approved by the Institutional Review Board of Yonsei University Dental Hospital (approval no. 2-2019-0026, granted on July 30, 2019). A real-time two-dimensional B-mode US system (E-CUBE 15 Platinum, ALPINION Medical Systems, Seoul, Korea) with a 60-mm-wide linear-array transducer (8.0–17.0 MHz; L8-17X, ALPINION Medical Systems) was used to obtain US images of the masseter muscle of healthy young individuals. These US images are unpublished data. The tables and figures in this paper were constructed based on data from the Supplementary Information.

### Participant selection and data acquisition

Signed written informed consent and facial US image data were obtained from 86 healthy, young individuals (48 males and 38 females, aged 25.4 ± 4.1 years). The exclusion criteria were orthodontic treatment, temporomandibular joint disorder, plastic surgery, or botulinum neurotoxin injection within the previous 6 months. The participants were placed in a supine position on a chair reclined at 45°. The US sampling frequency was adjusted to 15.0 MHz, which is an ideal frequency for observing depths between 1.5 and 4 cm, depending on the presence of skin, fat, and muscle tissues. The US transducer was positioned perpendicular to the skin surface over the scanning site. US scanning was performed on the midline and left side of the face. We used MATLAB deep-learning tools to implement the predictive model.

**Figure 1:**
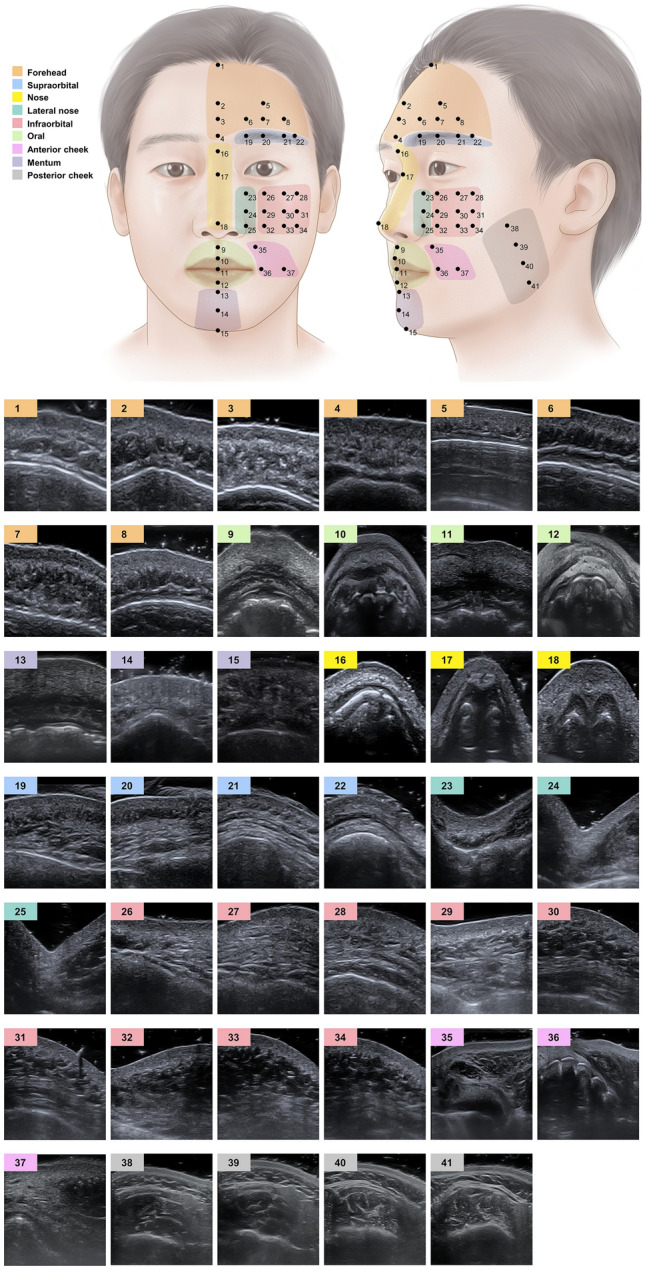
Nine facial regions, their landmarks, and US images corresponding to each landmark. Transverse US images at the region were used for deep learning models. **Forehead**: **1**, trichion (hair line at the midline); **2**, metopion (midpoint of bilateral frontal eminence), **3**, half point between 2 and 4; **4**, glabella; **5**, frontal eminence; **6**, meeting point between lines passing 3 and medial canthus; **7**, meeting point between lines passing 3 and mid-pupil; **8**, meeting point between lines passing through 3 and lateral canthus. **Oral**: **9**, half point between subnasale and 10; **10**, lower point on cupid’s bow; **11**, stomion; **12**, midpoint of lower vermillion border. **Mentum: 13**, deepest point of the chin at the midline; **14**, pogonion; **15**, gnathion. **Nose: 16**, sellion; **17**, rhinion; **18**, pronasale. **Supraorbital**: **19**, meeting point between lines passing 20 and the medial canthus; **20**, superior orbital rim at the mid-pupillary line; **21,** meeting point between lines passing 20 and the lateral canthus; **22**, meeting point between lines passing 20 and the lateral orbital rim. **Lateral nose**: **23**, meeting point between lines passing 26 and the medial canthus; **24**, point between 23 and 25; **25**, alare. **Infraorbital**: **26**, superior orbital rim at the mid-pupillary line; **27**, meeting point between lines passing 26 and the lateral canthus; **28**, meeting point between lines passing 26 and the lateral orbital rim; **29**, point between 26 and 32; **30**, point between 27 and 33; **31**, point between 28 and 34; **32**, meeting point between lines passing alare and middle pupil; **33**, meeting point between lines passing alare and the lateral canthus; **34**, meeting point between lines passing alare and the lateral orbital rim. **Anterior cheek**: **35**, meeting point between the line passing 9 and nasolabial folds; **36**, meeting point between lines passing stomion and middle pupil; **37**, meeting point between lines passing stomion and lateral cantus. **Posterior cheek**: **38–41**, points that divide the masseter by the upper and lower boundaries.

Deep learning models trained based on ImageNet data were evaluated for the classification of the nine facial regions. A total of 1440 US images were obtained from volunteers. From these, 160 US images were obtained from each region. All US images were transverse cross-section images. The facial landmarks and related US images for each facial region are shown in Fig. 1.

### CNN models for the classification of facial US images

ImageNet database, the most common and representative deep learning database, employed millions of images to train models and compared the classification performance of photographed facial US images. The evaluated CNN models were (1) GoogleNet, (2) SqueezeNet, (3) Mobilenet-v2, (4) ResNet-18, (5) ResNet-50, (6) ResNet-101, (7) Inception-v3, (8) Inception-ResNet-v2, (9) AlexNet, (10) VGG-16, (11) VGG-19, (12) DenseNet-201, (13) Xception, (14) NasNet-Mobile, and (15) ShuffleNet (Table 1).

**Table 1:** Pre-trained deep learning models using ImageNet.

Model	Depth	Size (MB)	Parameters (millions)	Image input size
AlexNet	8	227	61	227 × 227
DenseNet-201	201	77	20	224 × 224
GoogleNet	22	27	7	224 × 224
Inception-ResNet-v2	164	209	55.9	299 × 299
Inception-v3	48	89	23.9	299 × 299
Mobilenet-v2	53	13	3.5	224 × 224
NasNet-Mobile	^a^	20	5.3	224 × 224
ResNet-18	18	44	11.7	224 × 224
ResNet-50	50	96	25.6	224 × 224
ResNet-101	101	167	44.6	224 × 224
ShuffleNet	50	5.4	1.4	224 × 224
SqueezeNet	18	5.2	1.24	227 × 227
VGG-16	16	515	138	224 × 224
VGG-19	19	535	144	224 × 224
Xception	71	85	22.9	299 × 299

^a^The NasNet-Mobile does not consist of a linear sequence of modules. MB, megabyte.

### Verification of the nine regions of the face classification ability using the selected model

We trained 15 deep learning models to classify nine facial regions (Fig. 1). The training was conducted after adjusting the US image size to 224 × 224 × 3, 227 × 227 × 3, and 299 × 299 × 3 transforming the image to match the input size of the pre-trained deep learning model and augmenting the images. The training images were randomly translated up to 30 pixels and horizontally and vertically scaled up and down to 10%.

We evaluated the performance of each model using a tenfold cross-validation method. For the 160 US images of each region, 20 images were used as a test set, while the remaining 140 were divided into ten folds. One model has ten trained sub-models, and the sub-models were each evaluated for performance against the test set.

The training set for the model was a mini-batch size of 20, and the stochastic gradient descent with momentum (SGDM) moment was used. The maximum number of epochs was 20, and the learning rate was 0.0003, which was constant throughout the training.

### Evaluation metrics

#### Precision and recall

We calculated the precision by dividing the number of True Positive elements by the total number of positively predicted units, where “*k*” represents a generic class.

$${Precision}_{k}= \frac{{True \; Positive}_{k}}{{True \; Positive}_{k}+{False \; Positive}_{k}}$$

The recall was calculated by dividing the number of True Positive elements by the total number of positively classified units.

$${Recall}_{k} = \frac{{True \; Positive}_{k}}{{True \; Positive}_{k}+{False \; Negative}_{k}}$$

The arithmetic mean of the metrics for separate classes is used to calculate the Macro Average Precision and Recall, where *K* is the total number of class.

$$Macro\;Average\;Precision = \frac{{\sum }_{k=1}^{K}{Precision}_{k}}{K}$$

$$Macro\;Average\;Recall = \frac{{\sum }_{k=1}^{K}{Recall}_{k}}{K}$$

#### Accuracy

The accuracy was calculated by dividing the correct predictions (including true positives and true negatives) by the total number of examined cases.

$$Accuracy= \frac{True \;Positive+True \;Negative}{True \;Positive+True\; Negative+False \;Positive+ False \;Negative}$$

#### F-measure

F-measure or F1-Score aggregates Precision and Recall measures under the concept of harmonic mean was measured.

$${F\text{-}measure}_{k}=2\times \left(\frac{{Precision}_{k} \times {Recall}_{k}}{{Precision}_{k}+{Recall}_{k}}\right)$$

Macro F-measure, which is the arithmetic mean of class-wise F-measure, was calculated as shown below.

$$Macro \;F\text{-}measure = \frac{{\sum }_{k=1}^{K}{F\text{-}measure}_{k}}{K}$$

The performance of the deep learning model was evaluated using the abovementioned metrics, and the performance score of one model is the mean of tenfold scores. The score for the model training is provided as the final accuracy and loss value. The score for the validation set is shown as precision, recall, and F-measure. Each result is illustrated in tables and box plots.

### LIME (locally interpretable model-agnostic explanations)

Deep learning models are complicated, and their actions may be difficult to comprehend. The LIME approach approximates a deep neural network’s classification behavior with a smaller, more easily interpretable model^42^. The neural network’s decisions may be deduced by interpreting the decisions of this simpler model.

As the first step in the LIME method, we divided the ultrasound image into a grid of square features. The LIME method then uses bicubic interpolation to up-sample the computed map to match the image resolution. A 10 × 10 grid of features was created to increase the resolution of the computed map. LIME creates a composite image based on the original observation by randomly selecting a feature and replacing all pixels of that feature with the average image pixel, effectively removing that feature. The number of random samples was set to 6,000. The linear regression model used lasso regression.

### Facial US images’ quality

**Figure 2:**
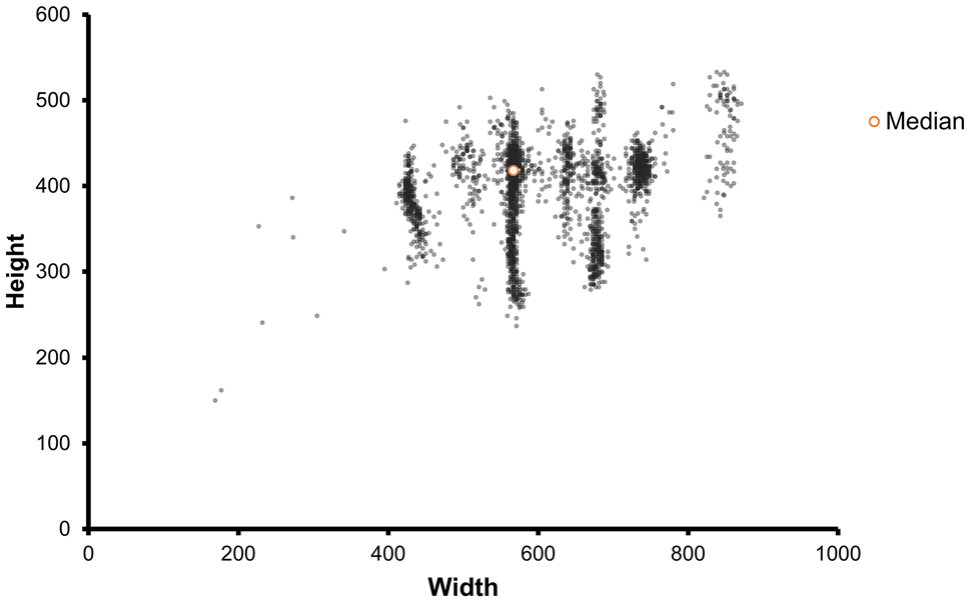
Scatter plot of facial US images’ size.

**Figure 3:**
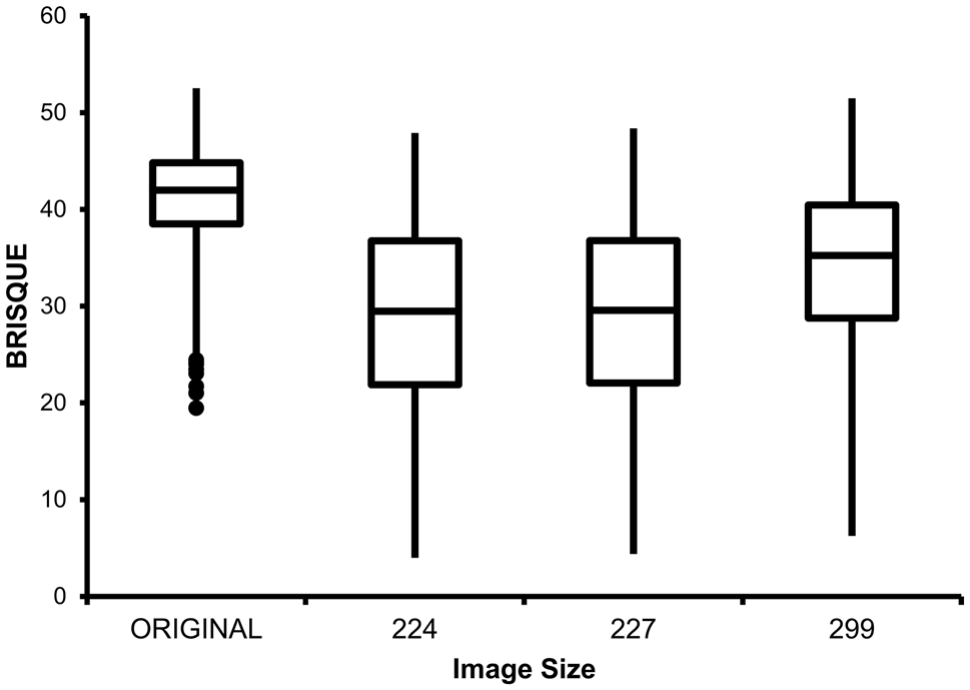
BRISQUE score according to facial US image size change. 224: 224 × 224 × 3, 227: 227 × 227 × 3, 229: 229 × 229 × 3.

The sizes of US images used in this study were 169 × 150 × 3 (smallest); 567 × 418 × 3 (medium); and 848 × 533 × 3 (largest) (Fig. 2). When US images of various sizes are transformed to fit the data input size of the deep learning model, the quality of the images changes. The quality of each transformed image and its original was quantified using BRISQUE (Blind/Referenceless Image Spatial Quality Evaluator) and displayed through a box plot (Fig. 3).

### BRISQUE

BRISQUE is an image analysis tool that adopts mathematical evaluation rather than objective image quality grading^43^. Unlike a qualitative comparison performed by humans, this is a repeatable quantitative method for image quality inspection. BRISQUE is a feature calculation model that simply employs picture pixels. It is shown to be highly efficient because it calculates its characteristics without the use of any transformations. According to the BRISQUE scoring system, the image quality values range from 0 to 100, corresponding to best and worst, respectively.

## Results

During the training process of all models, the accuracy and loss values reached a plateau between 10 and 15 epochs. All average values are arithmetic mean values and are shown with standard deviation.

### Training results of the models

**Figure 4:**
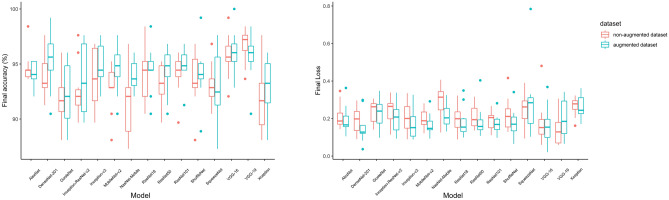
Training results for 10 folds of each deep learning model.

After training for ultrasound facial region classification, the mean of the final accuracy of all models using the non-augmented dataset was 93.56 ± 1.38%. The model with the lowest mean final accuracy of 91.50 ± 3.36% was NasNet-Mobile, while the model with the highest mean final accuracy was VGG-19 with 96.75 ± 1.60% (Table 2 and Fig. 4).

**Table 2:** The training final accuracy and loss values of the model using the non-augmented dataset and the models using the augmented dataset (accuracy: mean ± standard deviation %).

Model	Non-augmented dataset		Augmented dataset	
	Accuracy	Loss	Accuracy	Loss
AlexNet	94.68 ± 1.40	0.21 ± 0.06	94.12 ± 1.13	0.19 ± 0.07
DenseNet-201	93.88 ± 1.94	0.19 ± 0.07	95.31 ± 2.60	0.15 ± 0.08
GoogleNet	91.90 ± 2.14	0.23 ± 0.06	92.22 ± 3.03	0.23 ± 0.07
Inception-ResNet-v2	92.53 ± 2.45	0.24 ± 0.07	93.65 ± 3.26	0.20 ± 0.07
Inception-v3	93.73 ± 2.68	0.2 ± 0.07	94.92 ± 2.05	0.16 ± 0.07
Mobilenet-v2	92.77 ± 2.09	0.19 ± 0.04	94.52 ± 2.06	0.16 ± 0.05
NasNet-Mobile	91.50 ± 3.36	0.28 ± 0.08	93.88 ± 1.35	0.21 ± 0.06
ResNet-18	94.04 ± 2.40	0.19 ± 0.07	94.52 ± 2.13	0.18 ± 0.08
ResNet-50	93.17 ± 1.84	0.21 ± 0.06	94.36 ± 2.06	0.18 ± 0.08
ResNet-101	94.12 ± 1.80	0.18 ± 0.04	94.52 ± 1.88	0.17 ± 0.05
ShuffleNet	93.49 ± 2.50	0.22 ± 0.08	94.12 ± 2.54	0.17 ± 0.07
SqueezeNet	93.17 ± 1.80	0.24 ± 0.08	93.01 ± 3.05	0.28 ± 0.19
VGG-16	95.71 ± 1.98	0.17 ± 0.11	96.03 ± 2.01	0.16 ± 0.10
VGG-19	96.74 ± 1.60	0.13 ± 0.07	95.63 ± 2.15	0.20 ± 0.10
Xception	91.82 ± 2.89	0.26 ± 0.04	92.85 ± 2.84	0.25 ± 0.06

The lowest final accuracy among all folds was that of NasNet-Mobile, which recorded 87.30%, while the highest was 99.20%, recorded by the fold of NasNet-Mobile. The mean of the final loss values of all models was 0.22 ± 0.03. VGG-19 showed the lowest average loss value of 0.13 ± 0.07, and NasNet-Mobile revealed the highest average loss value of 0.28 ± 0.08. The model that recorded the lowest loss value among the folds was the fold of VGG-19 with a value of 0.06, while the fold that exhibited the highest loss value was that of VGG-16 with a value of 0.48 (Table 2 and Fig. 4).

The mean final accuracy was 94.25 ± 1.00% using the augmented dataset. The lowest mean final accuracy was recorded by GoogleNet as 92.22 ± 3.03%, and the highest one was recorded by VGG-16 as 96.03 ± 2.01%. The fold of SqueezeNet showed the lowest accuracy of 87.30% among the folds, while the model with the highest accuracy of 100% was that of VGG-16. The mean final loss values of all models was 0.19 ± 0.04. The DenseNet-201 model recorded the lowest average loss value, which was 0.15, while the highest average loss value was 0.28, recorded by SqueezeNet. The model that recorded the lowest loss value among all folds was the fold of VGG-16 with a value of 0.02, while the model showing the highest loss value was 0.78 with SqueezeNet. The mean of the lowest final accuracy among all models was recorded by GoogleNet as 92.22 ± 3.03%, and the model with the highest accuracy was VGG-16, recording 96.03 ± 2.01%. The fold of SqueezeNet showed the lowest accuracy among all folds at 87.30%, and the model with the highest accuracy of 100% was VGG-16. The mean of the final loss values of all models was 0.19 ± 0.04. The DenseNet-201 model recorded the lowest average loss value at 0.15, while SqueezeNet recorded the highest average loss value of 0.28. The model that recorded the lowest loss value among all folds was the fold of VGG-16 with a value of 0.02, while SqueezeNet was the model showing the highest loss value of 0.78 (Table 2 and Fig. 4).

### Test results of models

**Figure 5:**
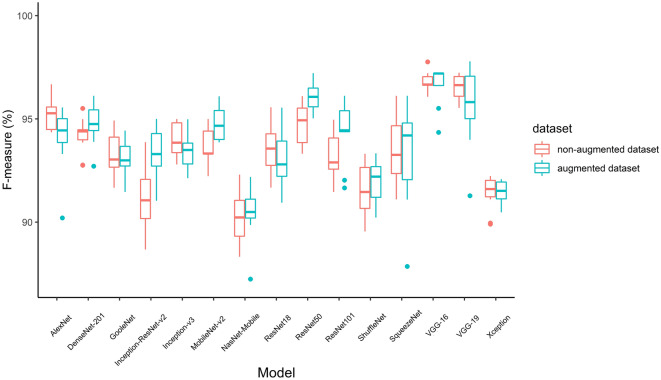
Test results for 10 folds of each deep learning model.

The mean values of precision, recall, and F-measure for the test set of all models using the non-augmented dataset were 93.88 ± 1.37, 93.55 ± 1.83%, and 93.52 ± 1.83%, respectively. The order of prediction, recall, and F-measure scores of the models was the same. The models with the lowest and highest scores were NasNet-Mobile and VGG-16, respectively. The fold scores suggestive of the lowest precision, recall, and F-measure were those of NasNet-Mobile, which were 89.11%, 88.33%, and 88.33%, respectively. The fold with the highest scores was the fold of VGG-16, with precision, recall, and F-measure scores of 97.80%, 97.78%, and 97.76%, respectively (Table 3 and Fig. 5).

**Table 3:** Performance on the test set of the model with the non-augmented dataset and the models with the augmented dataset (model) (mean ± standard deviation %).

Model	Non-augmented dataset			Augmented dataset		
	Precision	Recall	F-measure	Precision	Recall	F-measure
AlexNet	95.51 ± 0.74	95.22 ± 0.79	95.23 ± 0.79	94.51 ± 1.29	94.11 ± 1.64	94.12 ± 1.58
DenseNet-201	94.35 ± 0.88	94.22 ± 0.88	94.20 ± 0.87	94.99 ± 0.90	94.72 ± 0.95	94.74 ± 0.98
GoogleNet	93.65 ± 1.15	93.28 ± 1.06	93.24 ± 1.06	93.58 ± 0.74	93.06 ± 0.92	93.00 ± 0.96
Inception-ResNet-v2	91.70 ± 1.31	91.17 ± 1.45	91.13 ± 1.49	93.74 ± 1.22	93.33 ± 1.31	93.30 ± 1.33
Inception-v3	94.24 ± 0.82	94.00 ± 0.86	93.96 ± 0.85	93.86 ± 0.99	93.56 ± 0.99	93.48 ± 1.00
Mobilenet-v2	93.92 ± 0.84	93.67 ± 0.88	93.66 ± 0.86	95.03 ± 0.80	94.83 ± 0.87	94.80 ± 0.87
NasNet-Mobile	91.06 ± 1.27	90.17 ± 1.28	90.13 ± 1.30	91.32 ± 1.17	90.44 ± 1.30	90.41 ± 1.30
ResNet-18	93.82 ± 1.12	93.50 ± 1.17	93.49 ± 1.17	93.58 ± 1.29	94.44 ± 1.39	93.04 ± 1.44
ResNet-50	95.07 ± 0.97	94.83 ± 1.02	94.79 ± 1.01	96.31 ± 0.62	96.06 ± 0.67	96.05 ± 0.66
ResNet-101	93.71 ± 1.00	93.33 ± 1.14	93.20 ± 1.18	94.97 ± 1.14	96.06 ± 1.48	94.41 ± 1.51
ShuffleNet	92.06 ± 1.18	91.56 ± 1.36	91.59 ± 1.30	92.52 ± 0.94	91.94 ± 1.12	91.98 ± 1.09
SqueezeNet	93.95 ± 1.39	93.50 ± 1.53	93.47 ± 1.52	93.86 ± 1.86	93.28 ± 2.33	93.24 ± 2.44
VGG-16	96.88 ± 0.50	96.78 ± 0.51	96.76 ± 0.51	96.85 ± 0.84	96.67 ± 0.94	96.64 ± 0.97
VGG-19	96.69 ± 0.57	96.56 ± 0.57	96.54 ± 0.58	95.99 ± 1.68	95.56 ± 1.98	95.58 ± 1.92
Xception	91.63 ± 0.77	91.44 ± 0.84	91.39 ± 0.84	91.71 ± 0.57	91.56 ± 0.63	91.44 ± 0.59

The precision score for region classification was lowest in the oral region at 87.85 ± 5.35%, followed by the orbit-upper region at 87.97 ± 7.36%. The recall score was lowest in the anterior cheek at 82.3 ± 6.33%. The F-measure scores were lowest in the anterior cheek and orbit-upper regions at 87.31 ± 4.11% and 87.71 ± 5.48%, respectively. The regions with the highest precision, recall, and F-measure scores were the lateral nose and nose regions. Precision and F-measure scores were 99.8 ± 0.93% and 99.11 ± 1.21% in the lateral nose region, and the recall score was highest in the nose region (98.73%) (Table 3 and Fig. 5).

The mean values of precision, recall, and F-measure for the test set of all models using the augmented dataset were 94.18 ± 1.53, 93.77 ± 1.63%, and 93.74 ± 1.65%, respectively. The order of precision, recall, and F-measure scores of the models were all the same. The model with the lowest score was NasNet-Mobile, while the model with the highest score was VGG-16. The fold scores indicative of the lowest precision, recall, and F-measure were those of NasNet-Mobile, which were 88.72%, 87.22%, and 87.23%, respectively. The highest fold scores were those of the VGG-19 fold, which were 97.85%, 97.77%, and 97.79%, respectively (Table 3 and Fig. 5).

**Figure 6:**
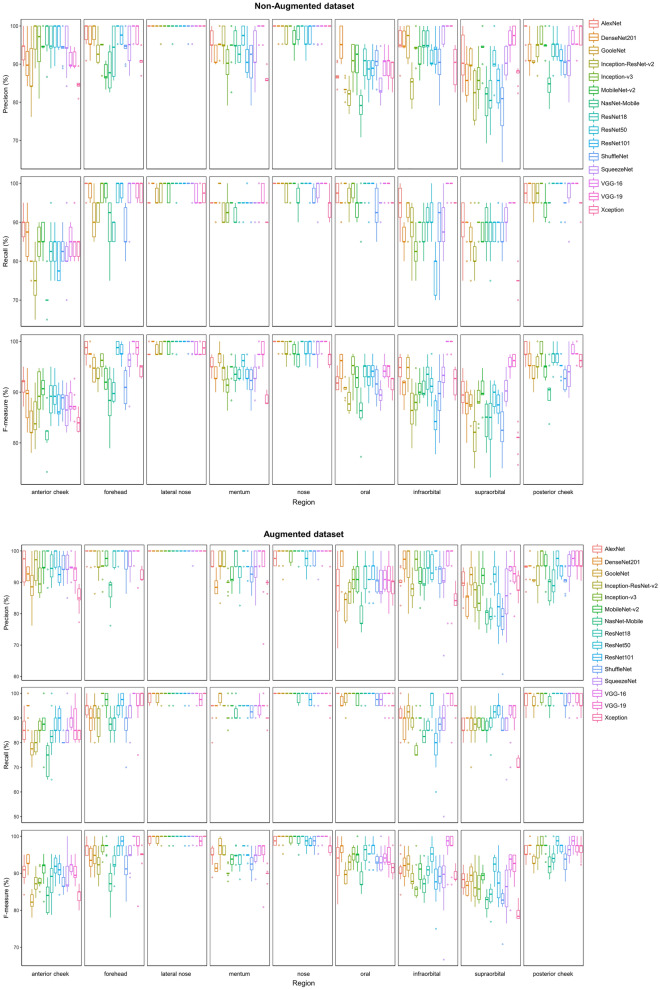
Test results for 10 folds of each deep learning model in each region.

The precision score for region classification was lowest in the orbit-upper region at 86.77 ± 7.58%, followed by that of the oral region, which was 89.31 ± 6.36%. The recall score was lowest in the anterior cheek at 84.5 ± 7.25%. The F-measure scores were the lowest in the anterior cheek and the orbit-upper regions at 88.64 ± 4.37% and 86.84 ± 5.14%, respectively. The lateral nose region exhibited the highest precision, recall, and F-measure scores, which were 99.93 ± 0.54%, 99.33 ± 1.7%, and 99.62 ± 0.9%, respectively (Table 4 and Fig. 6).

**Table 4:** Performance on the test set of the model with the non-augmented dataset and the models with the augmented dataset (region) (mean ± standard deviation %).

Region	Non-augmented dataset			Augmented dataset		
	Precision	Recall	F-measure	Precision	Recall	F-measure
Anterior cheek	93.54 ± 5.59	82.30 ± 6.33	87.31 ± 4.11	93.82 ± 5.3	84.5 ± 7.25	88.64 ± 4.37
Forehead	95.03 ± 5.01	95.50 ± 5.54	95.13 ± 4.06	96.90 ± 4.52	93.23 ± 6.29	94.88 ± 4.23
Lateral nose	99.80 ± 0.93	98.46 ± 2.31	99.11 ± 1.21	99.93 ± 0.54	99.33 ± 1.70	99.62 ± 0.90
Mentum	93.88 ± 4.96	94.30 ± 2.89	94.01 ± 3.13	94.31 ± 5.26	94.00 ± 3.17	94.05 ± 3.10
Nose	98.95 ± 2.18	98.73 ± 2.6	98.81 ± 1.69	99.08 ± 2.08	99.26 ± 1.95	99.15 ± 1.43
Oral	87.85 ± 5.35	96.93 ± 3.56	92.05 ± 3.41	89.31 ± 6.36	98.56 ± 2.54	93.56 ± 3.57
Infraorbital	94.13 ± 5.45	90.13 ± 7.16	91.88 ± 4.97	93.33 ± 6.3	89.2 ± 8.33	90.83 ± 5.14
Supraorbital	87.97 ± 7.36	87.86 ± 6.24	87.71 ± 5.48	86.77 ± 7.58	87.56 ± 6.67	86.84 ± 5.14
Posterior cheek	93.74 ± 4.72	97.7 ± 3.2	95.6 ± 3.09	94.14 ± 4.43	98.26 ± 2.95	96.09 ± 2.84

## Discussion

For facial ultrasound image region classification, the relatively classic models VGG-16, VGG-19, and ResNet-50 had the highest scores (Table 3 and Fig. 5). Looking at the above simplification, the models with better performance have in common a large number of parameters, shallow depth, and small image input size (Table 1 and Table 3). The same was observed in previous studies when comparing deep learning performance on medical images such as ultrasound and CT images, where shallow and classical models performed better than deep modern algorithms^44^. Considering that the performance is improved from ResNet-18 to ResNet-50 and then decreased in ResNet-101, it seems that a numerical balance between the model depth and the number of parameters is necessary.

The BRISQE score for US images generally shows the highest score among medical images such as MRI and CT, indicating the lowest image quality^34^. Counterintuitively, the BRISQUE score tended to decrease as the US image size was, arbitrarily, reduced in this study. This may be related to the high-performance scores of the models using the small US image size.

The average performance of the model using the augmented dataset was 0.2% higher than the model using the non-augmented dataset; therefore, there was no significant difference in the performance of the model regardless of whether the data was augmented or not. A significant performance improvement was exceptionally observed only in Inception-ResNet-v2, ResNet-50, and ResNet-101 among the 15 models evaluated in this study (Table 3 and Fig. 5). Data augmentation is the most popular method implemented to prevent overfitting^45^. The dataset was augmented by horizontal movement and zoom in and out according to the characteristics of neighboring landmarks in the region used in this study; however, the effect was weak. This indicates that the effect of data augmentation may vary depending on data characteristics or models. As in the case of Inception-v3, the performance score decreased after augmentation in some cases; thus, training using unconditional data augmentation requires attention.

**Figure 7:**
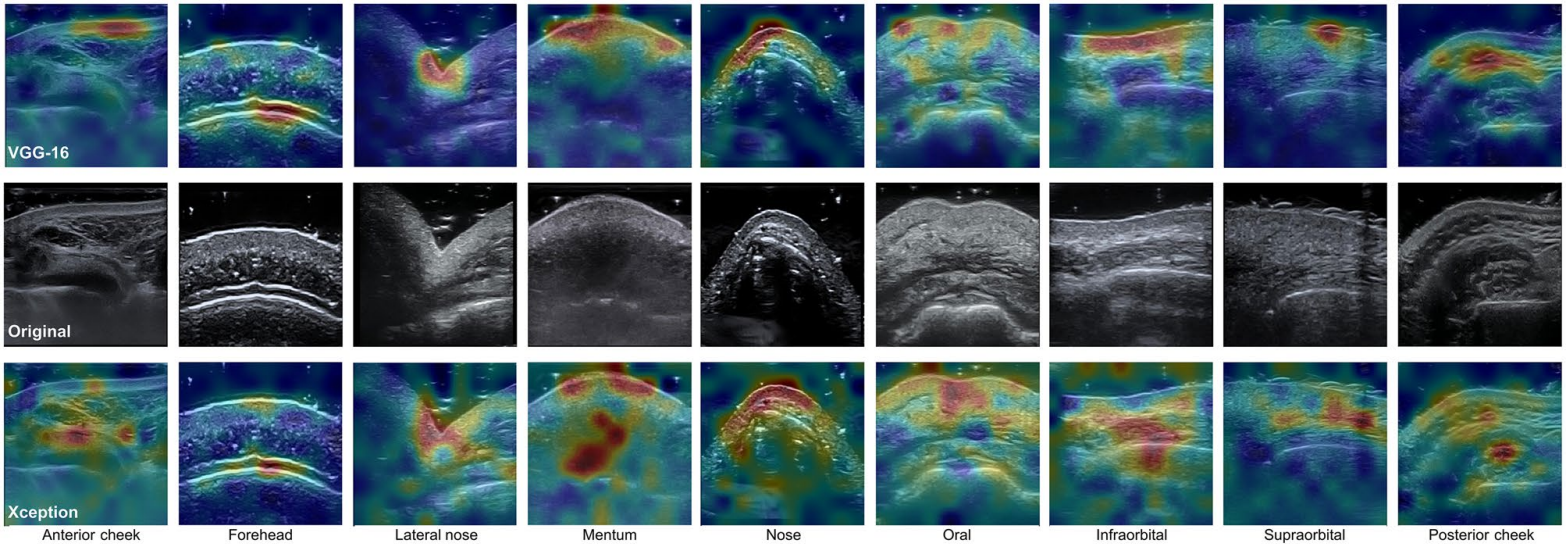
LIME analysis of classification criteria for the facial region of VGG-16 and Xception. The top row is the local features considered by VGG-16, the middle row is the original image, and the bottom row is the local features considered by Xception. The red area is the strongly weighted local, and the blue area is the weakly weighted local.

The average performance score for each region was about 85% to 99%, which significantly differed between each region. Among all regions, lateral nose and nose were the most clearly distinguished (Table 4 and Fig. 6). By examining the most meaningful locals in the lateral nose and nosed through LIME, it is evident that the models clearly distinguish the skin and bone contours from other regions and their features (Fig. 7). Although the shape of the other regions under investigation are different, the models mainly considered hyperechoic skin and bones or their surroundings as the main features. Artifacts such as gels and bone shadows were sometimes regarded by the models as genuine features; however, in most cases, the artifacts were suitably ignored.

Irrespective of the model, the local features of each region viewed with LIME were similar. The VGG models had exceptionally high-performance scores in the orbital-lower and orbital-upper regions, and the attention areas of VGG models examined through LIME were the smallest compared to the areas of other models. This tendency seems to be a reason why VGG models have lower performance than other models in the case of anterior cheeks. Mentalis m. and masseter m., which are relatively hypoechoic areas of the muscles in the mentum and posterior cheeks, were ignored. Moreover, the model that considered these muscles as the main features showed rather poor performance.

When segmentation is performed on a facial ultrasound image, the structure shown by each region is very different; thus, it is critical to label each region separately. If segmentation is performed without pre-classifying the face parts in this manner, many images are expected to be required to achieve proper performance. Recently, methods to improve the performance of the segmentation model by combining the feature maps of each stage in the segmentation model encoder and the classification model have been introduced^46^.

In conclusion, the quality and characteristics of the input data are a significant part of deep learning training, and in the case of training using a small number of data, it responds sensitively. The repetition of a structure with clear contrast on the US image in one class during transfer education using a model pre-trained with ImageNet is expected to have a significant impact on feature extraction. When conducting transfer education using a small number of images, it seems crucial to properly filter the US image and strengthen the contrast for the main structures. In deep learning models, muscles, blood vessels, and nerves that lack contrast in the segmentation of facial US images appear to be easily ignored. In the poor-quality US images’ characteristic, the classical deep learning model showed better classification performance. Since the analysis through LIME is limited to local analysis, it was difficult to compare models with little performance difference. For detailed performance comparison, a method that can perform global analyses is required. The results of this study can be used as reference data for future deep learning research on facial US images and content development (Supplementary Information).

## Supplementary Information

Supplementary Information.

## Data Availability

The datasets used and/or analyzed during the current study are available from the corresponding author on reasonable request. All data generated or analyzed during this study are included in this published article.

## Abbreviations

MRI: Magnetic resonance imaging
CT: Computed tomography
US: Ultrasonography
LIME: Locally interpretable model-agnostic explanations
BRISQUE: Blind/Referenceless Image Spatial Quality Evaluator
